# Assessment of the Natural Radioactivity of Polish and Foreign Granites Used for Road and Lapidary Constructions in Poland

**DOI:** 10.3390/ma13122824

**Published:** 2020-06-23

**Authors:** Tomasz Drzymała, Aneta Łukaszek-Chmielewska, Sylwia Lewicka, Joanna Stec, Barbara Piotrowska, Krzysztof Isajenko, Paweł Lipiński

**Affiliations:** 1 Faculty of Safety Engineering and Civil Protection, The Main School of Fire Service, 52/54 Słowackiego Str., 01-629 Warsaw, Poland; tdrzymala@sgsp.edu.pl (T.D.); alukaszek@sgsp.edu.pl (A.Ł.-C.); stecjoann@gmail.com (J.S.); 2 Central Laboratory for Radiological Protection, 7 Konwaliowa Str., 03-194 Warsaw, Poland; piotrowska@clor.waw.pl (B.P.); isajenko@clor.waw.pl (K.I.); p.lipinski@clor.waw.pl (P.L.)

**Keywords:** granite, building materials, natural radioactivity, absorbed dose, effective dose, uranium, radium, potassium

## Abstract

The measurements of the specific activity of natural radioactive isotopes of radium (^226^Ra), thorium (^232^Th) and potassium (^40^K) in chosen samples of imported (China, Finland, Spain, India, Sweden) and Polish (Izerski, Karkonosze, Siedlimowicki, Strzegomski, Strzelinski) granites were performed. The measurements were carried out with 2 × 2” NaI(Tl) scintillation detector. The measured specific activity on natural radioactive isotopes were within the following ranges: 5.8–312 [Bq kg^−1^], 5.5–189 [Bq kg^−1^] and 109–1590 [Bq kg^−1^] for ^226^Ra, ^232^Th and ^40^K, respectively. Obtained concentrations of radioactive isotopes allowed to perform the analysis of the exposure of the humans from the ionizing radiation emitted by the granites. The determination of the exposure consisted in the calculation of absorbed gamma dose rate (D) [nGy h^−1^] for each sample, which fell in the range between 20 and 511 [nGy h^−1^], annual effective dose rate (AED) [mSv year^−1^] ranging between 0.10 and 2.50 [mSv year^−1^], radium equivalent activity (Ra_eq_) [Bq kg^−1^] with values between 22 and 570 [Bq kg^−1^], external and internal hazard indices (H_ex_) and (H_in_) falling in the ranges 0.06−1.53 and 0.08–2.41 respectively, as well as gamma (I_γ_) and alpha (I_α_), representative level indices with values 0.08–2.0 and 0.029–1.56, respectively. Moreover, obtained results were compared with the international standard values given by the European Commission (EC), the United Nations Scientific Committee on the Effects of Atomic Radiation given in UNSCEAR Reports, and the results of research from other laboratories.

## 1. Introduction

The granites exhibit very good technical and chemical properties and are primarily valued for their esthetical quality. They are exceptionally resistant to frost, weathering and abrasion, while maintaining perfect grinding and polishing properties. Thus, due to these properties, the granite rocks are widely used in bridge, road, water and lapidary constructions, mainly as construction elements, monuments, road and pavement components [1]. 

Granite is magmatic igneous rock formed during the crystallization process of liquid magma inside the earth under high temperature and pressure. Granite is one of the most widely occurring rocks in the earth crust. It mainly consists of quartz with intrusions of potassium feldspar, as well as acidic plagioclase and mica. Quartz has white or grey grains, sometimes red or bluish. Moreover, the quartz grains are the final product of magma crystallization resulting in its irregular shapes. The grains of quartz usually fill the empty spaces between other minerals. The potassium feldspar appears in form of white-greyish, pink or red grains, which influences the color of granite. Both minerals (quartz and feldspar) can be distinguished by the hardness and differences in cleavage. Quartz is harder and non-cleavable, and potassium feldspars are characterized by distinct bidirectional cleavability, thus the grains of feldspar reflect the light by plain surfaces. Plagioclase is usually white or grey, sometimes red (due to the presence of iron oxide). The most common mica in granites is biotite, which is easily recognizable due to its appearance in form of dark, almost black lamellae having perfect cleavability [2,3,4,5]. 

The natural radioactivity of rocks and minerals is a common phenomenon. The radioactive isotopes of ^87^Rb, ^40^K, uranium-radium series and thorium series elements, as well as ^147^Sm, ^138^La, ^187^Re are present in the lithosphere [6]. The average concentration of potassium in the earth crust is about 2.6%, and of uranium and thorium 1.8 ppm and 7.2 ppm, respectively. The magmatic rocks, including granite, are, according to research by Mason and Moore [7], enhanced with uranium and thorium. The total content of uranium and thorium in granite rocks is on the average 5 and 15 ppm, and of potassium 5%, which is connected with the presence of potassium feldspar in granite [8]. The subject of the presence of radioactive isotopes in the earth crust is one of the most important geochemical research. Systematic measurements of the concentration of natural radioactive isotopes (i.e., radium, uranium, thorium and potassium) in rocks and minerals performed in many countries proved that acidic magmatic rocks (such as granite) are characterized by the highest radioactivity. Certain sedimentary rocks, i.e., clay and silt accompanying brown coal deposits, demonstrate similar level of radioactivity. Performing the research on the distribution and concentration of the radioactive isotopes in the earth crust it is important to take into account that the radioactive elements are dispersed and inhomogeneous in the rocks of the same type. For example, the quantity of radium in the Finland granites is twice when compared with granites from USA, Canada, Ireland or Japan, and it is even 4 to 6 times higher than in Antarctic granites [6]. The research on granites in Poland was performed by S. Pieńkowski, M. Borkowska, Z. Gryglewicz and C. J. Szwacka [9]. They observed that Karkonosze (Giant Mountains) granites and Kudowa massif granites are more abundant in radioactive elements than Strzelin massif granites and Strzegom-Sobotka granites [9]. Moreover, M. Wichrowska, Z. Wichrowski, I. Żejmo performed the research on radioactivity of biotites extracted from granitoids of Strzegom-Sobotka and Strzelin massifs [6,10]. D. Malczewski, A. Sitarek, J. Żaba, J. Dorda deal with the radioactivity of crystalline rocks of Izerski massif including Izerski and Rumburski granites [11]. They performed the measurements of the radioactivity of uranium, radium, thorium, potassium and others. The average concentration of potassium in their granite samples was 2.92% (2.95% Rumburski granite and 2.89% Izerski granite), and was lower than the figures in literature Eisenbud and Gesell [12]. Relatively low concentrations of uranium were measured: 2.66 ppm for Rumburski granite and 2.54 ppm for Izerski granite. These values are definitely lower than those recorded for Kudowa granites—3.94 ppm, Strzegom—7.31 ppm [13], but they are close to the average concentrations of uranium in continental crust granites, i.e., 3 ppm [12]. The specific activity of ^226^Ra isotope of the Rumburski and Izerski granites amounted to 33.1 Bq·kg^−1^ and 31.6 Bq·kg^−1^, respectively. The activity concentration of ^232^Th was equal 9.9 Bq·kg^−1^ and 11.8 Bq·kg^−1^ in Rumburski and Izerski granites, respectively. These concentrations are much lower than those measured for Kudowa granites—16.9 ppm, Strzegom—18.7 ppm, or Szklarska Poręba—32 ppm [13], at average thorium concentration in granites of 17 ppm [12]. 

Since granites are more and more popular as construction materials [14], the measurement of the radioactivity concentration of natural radioactive isotopes is especially important in this case, due to the need of the assessment of the exposure to the ionizing radiation (in particular for stonemasonry workers). Moreover, there is still few data on radioactivity of polish granites in the literature. Therefore, there has been a need to perform such experiments and to compare the radioactivity of polish granites to the world’s data. Based upon the measured concentrations of natural radioactive isotopes the radiological parameters such as absorbed gamma dose rate, (D), annual effective dose rate (AED), radium equivalent activity (Ra_eq_), external and internal hazard indices (H_ex_) and (H_in_), as well as gamma and alpha indices (I_γ_) and (I_α_,) were determined. The obtained results were compared with the average values recommended by the European Commission (EC) and the United Nations Scientific Committee on the Effects of Atomic Radiation given in UNSCEAR Reports, and the results of research from other laboratories.

## 2. Sample Preparation for Gamma Spectrometry 

Five Polish granites (Izerski, Karkonosze, Siedlimowice, Strzegom and Strzelin) commonly used for road construction and five imported granites (Europe—Spain, Sweden and Finland; Asia—China, India) used mainly as gravestones, were analyzed during this assessment. 

**Figure 1:**
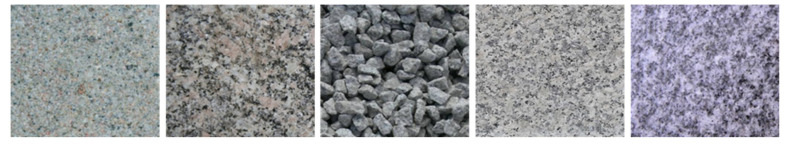
Successively: Izerski Granite, Karkonosze Granite, Siedlimowice Granite, Strzegom Granite, Strzelin Granite.

The granite massifs in Poland are present mainly in Lower Silesia, including Izerskie Mountains massif, Karkonosze (Giant Mountains) massif, Strzelin-Otmuchow, Strzegom-Sobotka and in the vicinity of Siedlimowice, Figure 1. All Lower-Silesian granites are true granites consisting of: quartz, potassium feldspar, plagioclase and mica (particularly biotite). They have granite grained structure [1].

**Figure 2:**
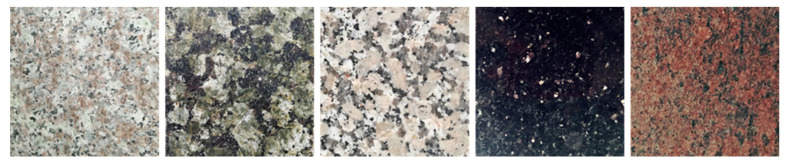
Successively: China (Royal Bronze), Finland (Baltic Green), Spain (Crema Julia), India (Star Galaxy), Sweden (Vanga).

Imported granites differ from domestic ones by color, as well as by shape of crystals, which is usually irregular. They are valued primarily for their unique esthetic appearance, Figure 2. 

Before performing the gamma spectrometry, all granite samples were crushed to form the 2 mm fraction. The first step consisted in crushing of the granite plates with pneumatic hammer to the size allowing for further preparation of the samples in the porcelain grinder. After crushing in the grinder, the granite was placed in the ball mill for 24 h. The samples were then poured into porcelain dishes and dried in a chamber drier at 100 °C for 24 h until a constant sample weight was achieved. The harder pieces that could not be broken with a hammer were broken in jaws and crushed in a crusher. The dried dust was sieved through 2 mm sieves. This procedure assures quite good homogeneity of the samples for testing the radioactivity concentrations, [15,16,17,18,19,20]. The samples prepared in this way were then poured into 1.5 dm^3^ cylindrical Marinelli beakers, then placed under the spectrometer probe. This approach is consistent with IAEA recommendations for environmental monitoring, [19]. The beakers were filled in such a way that the granite was 5 mm in front of its upper edge. Then the net mass of each of the prepared samples was measured. The concentration of radon (^222^Rn) was determined based upon the activity of radium (^226^Ra) progenies and for this purpose it was necessary to ensure the tightness of Marinelli containers to avoid losses of radon concentration which is a volatile product of radium (^226^Ra) decay. The concentration of thorium ^232^Th was determined based upon the measurement of the concentration of thallium ^208^Tl. Therefore, the samples were tested only after the radioactive equilibrium was established between ^226^Ra and ^214^Bi, as well as ^232^Th and ^208^Tl, i.e., after 4 weeks. The gamma ray spectra had been collected for eight hours for each sample, and the results for all the natural radionuclides have been obtained at the same time.

## 3. Gamma-Ray Spectrometry

The measurement of granites was performed using the MAZAR type spectrometer (Warsaw, Poland) connected with 2 × 2” NaI(Tl) scintillation detector. To minimize the radiation background the scintillation probe was placed in the lead chamber with 50 mm thick walls. MAZAR spectrometer is an analyzer working in three measuring ranges, which allow determining the concentrations of the following radioactive isotopes: ^40^K, ^226^Ra and ^232^Th. Specific measurement channels include the following energy ranges of gamma photons:

(1) Channel 1, with energy range 1.26–1.65 MeV, detects photons emitted by the isotope of potassium (^40^K) having the energy of 1.46 MeV, also photons belonging to the Compton spectra of gamma rays of radioisotopes of the thorium and uranium series, as well as the background gamma radiation and the intrinsic background of the spectrometer;
(2) Channel 2, with energy range 1.65–2.30 MeV, detects photons emitted by the isotope of bismuth (^214^Bi) having the energy of 1.76 MeV in equilibrium with ^226^Ra radium isotope, also Compton spectrum from thallium ^208^Tl isotope, as well as the background gamma radiation and the intrinsic background of the spectrometer;
(3) Channel 3, with energy range 2.30–2.85 MeV, detects photons emitted by the isotope of thallium (^208^Tl) having the energy of 2.62 MeV in equilibrium with thorium (^232^Th), and again, the background gamma radiation and the intrinsic background of the spectrometer [21].

The detector efficiency calibration was based on the measurements of three volumetric calibration standards: ^40^K, ^226^Ra and ^232^Th and the measurement of the matrix of standards as a background measurement, which results in obtaining ten calibration coefficients calculated by the matrix method. The geometry of reference sources and tested samples were Marinelli beakers with volume of 1.5 dm^3^. The density of volumetric calibration standards was 1.60 g·cm^−3^, while the bulk density of the measured samples was in the range between 0.83 and 1.69 g·cm^−3^. The energy resolution of the spectrometer was 6–8%. During every experiment, the spectrometer detected gamma photons, which were counted in the dedicated program. The activity concentration was then determined for all the radioisotopes from one spectrum per a sample.

## 4. Radiological Parameters 

Knowledge about the concentrations of natural radioactive isotopes ^226^Ra, ^232^Th and ^40^K in the tested granite samples is necessary to determine the radiological hazard parameters in order to assess exposure of public to ionizing radiation from the above radioisotopes. 

The absorbed gamma dose rate in the air was calculated using the appropriate conversion coefficients of 0.92, 1.1 and 0.08 for radium (^226^Ra), thorium (^232^Th) and potassium (^40^K), respectively. The absorbed gamma dose rate is expressed by Equation (1), and according to the UNSCEAR 2000 Report this amount should not exceed indoors 84 nGy h^−1^ [22]

(1) $$D\;\left[\mathrm{nGy}\cdot h^{-1}\right]=0.92{\;S}_{\mathrm{Ra}}+1.1{\;S}_{\mathrm{Th}}+0.08{\;S}_{K}$$

where:

D—absorbed gamma dose rate [nGy h^−1^]

S_K_, S_Ra_, S_Th_—activity concentration of potassium, radium and thorium [Bq kg^−1^].

The annual effective dose rate is defined as the product of the absorbed dose rate, rounded number of hours per year and conversion coefficient from absorbed gamma dose rate into effective dose, equal to 0.7 × 10^−6^, see Equation (2). According to the UNSCEAR 2000 Report, the annual effective dose should read: AED ≤ 1 mSv·a^−1^ [22].

(2) $$\mathrm{AED}\left[\mathrm{mSv}\cdot a^{-1}\right]=D\left[\mathrm{nGy}\cdot h^{-1}]\cdot 7000h\cdot 0.7[\mathrm{Sv}\cdot {\mathrm{Gy}}^{-1}\right]\cdot 10^{-6}$$

where:

AED—annual effective dose rate [mSv a^−1^], a—annum (year), D—absorbed gamma dose rate [nGy h^−1^].

Gamma radiation hazards arising from specific radionuclides are assessed, based on the radium equivalent activity and external hazard index, calculated according to Equations (3) and (4), respectively. According to UNSCEAR 1982 Report it was assumed that Ra_eq_ should be less than or equal to 370 Bq kg^−1^, and H_ex_ less or equal to 1 [23].

Radium equivalent activity:

(3) $${\mathrm{Ra}}_{\mathrm{eq}}\;\left[\mathrm{Bq}\cdot {\mathrm{kg}}^{-1}\right]={\;S}_{\mathrm{Ra}}+1.43{\;S}_{\mathrm{Th}}+0.077{\;S}_{K}$$

External hazard index [24]:

(4) $$\mathrm{Hex}=\frac{S_{\mathrm{Ra}}}{370}+\frac{S_{\mathrm{Th}}}{259}+\frac{S_{K}}{4810}$$

In the course of research, the use of the gamma representative level index for building and construction materials, among others, should be determined and indicated, which for granite should be less than 1. The index refers to the gamma dose which, as a result of typical outdoor exposure, also occurs in buildings constructed of a particular type of building material. 

Gamma index is derived from the following Equations [25]:

(5) $$I\;=\frac{S_{\mathrm{Ra}}}{300\;\left[{\mathrm{Bq}\;\mathrm{kg}}^{-1}\right]}+\frac{S_{\mathrm{Th}}}{200\left[{\mathrm{Bq}\;\mathrm{kg}}^{-1}\right]}+\frac{S_{K}}{3000\;\left[{\mathrm{Bq}\;\mathrm{kg}}^{-1}\right]}$$

It is a non-dimensional quantity, and factors S_K_, S_Ra_ and S_Th_ were defined with reference to Equation (1).

Excess alpha radiation caused by inhalation of radon, which is a decay product of radium ^226^Ra, is estimated on the basis of the alpha index I_α_, see Equation (6). Recommended upper value of radon concentration, inside new buildings, is 200 [Bq m^−3^]. Therefore, when I_α_ exceeds the value of 1, then it would be possible that the concentration of radon released from this material might exceed the recommended value [26,27]. The formula reads as follows:

(6) $$I\alpha \;=\frac{S_{\mathrm{Ra}}}{200\;\left[{\mathrm{Bq}\;\mathrm{kg}}^{-1}\right]}$$

Internal hazard index is determined according to Equation (7):

(7) $$Hin\;\left[-\right]=\frac{S_{Ra}}{185}+\frac{S_{Th}}{259}+\frac{S_{K}}{4810}$$

The index determines the exposure of living organisms to internal radiation from radon ^222^Rn and its short-lived decay products, which are particularly dangerous for respiratory system. The level of the index shall not be higher than 1, so that the hazard from radon and its short-lived decay products is then insignificant and negligible [24].

## 5. Results and Discussion

**Figure 3:**
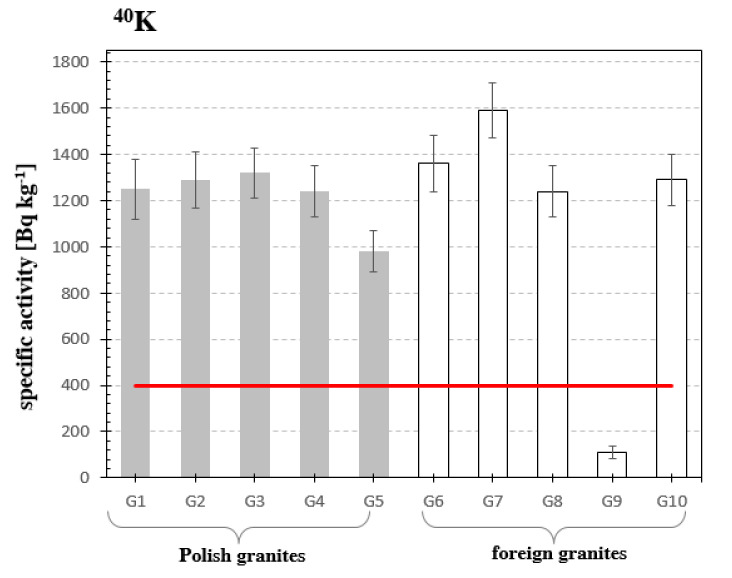
Specific activity of potassium ^40^K in analyzed samples of granites. The horizontal line indicates the average specific activity of ^40^K of building materials recommended by the European Commission in the Report [25] of 400 Bq·kg^−1^.

Analyzing the obtained results, it can be stated that the presence of potassium ^40^K isotope in all examined granite samples was detected. The concentrations of potassium isotope were in the range between 109 Bq·kg^−1^ for granite sample from India (G9) up to 1590 Bq·kg^−1^ for granite sample from Finland (G7). Taking into account average concentration values recommended by the European Commission [19] it can be seen that in all samples except G9, the concentration of ^40^K exceeded the recommended values many times. Based upon the research, it can be concluded that the use of granites as building materials causes an increase of the ionizing radiation level in relation to the natural background, see. Figure 3 and Table 1, which is also a summary table of all concentrations of measured isotopes with its uncertainties. As one can see, the uncertainties fall into the range between few to several dozens % of the result of the measurement.

**Figure 4:**
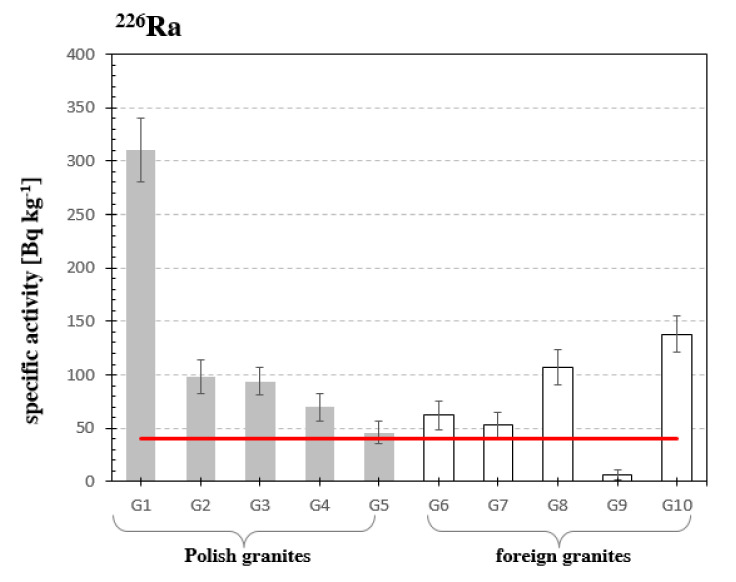
Specific activity of radium ^226^Ra in analyzed samples of granites. The horizontal line indicates the average specific activity of ^226^Ra of building materials recommended by the European Commission in the Report [25] of 40 Bq·kg^−1^.

The activity concentration of radium ^226^Ra of the measured samples of granite ranged from 5.8 Bq·kg^−1^ to 313 Bq·kg^−1^. The lowest activity concentration of radium amounting to 5.8 Bq·kg^−1^, was measured for the sample from India G9, and the highest for granite from Izerski granite G1, amounting to 313 Bq·kg^−1^, see Figure 4. Taking into account the average activity concentration of radium—40 Bq·kg^−1^ [25], it can be stated that almost all granites have a concentration of this element two or even three times higher, with the exception of Izerski granite G1, which the average recommended value exceeds about eight times. 

**Figure 5:**
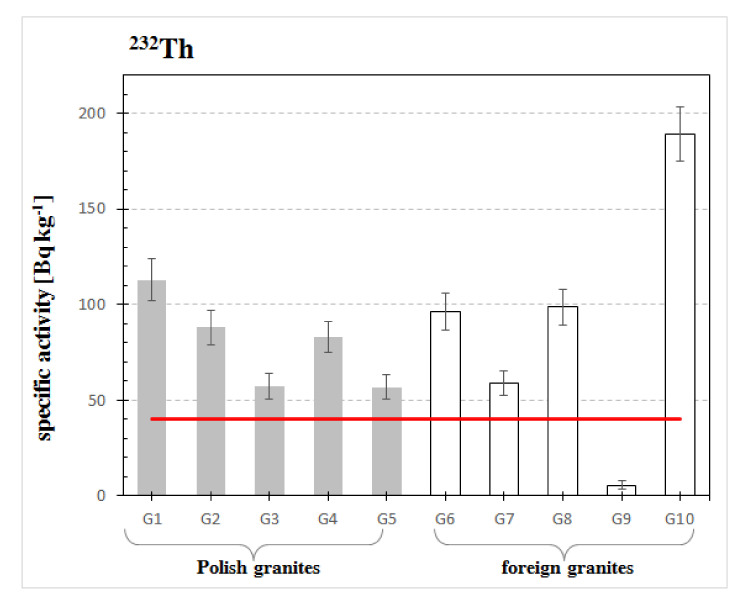
Specific activity of thorium ^232^Th in analyzed samples of granites. The horizontal line indicates the average specific activity of ^232^Th of building materials recommended by the European Commission in the Report [25] of 40 Bq·kg^−1^.

The range of the specific activity concentration of thorium isotope in the analyzed rocks was between 5.5 and 189 Bq·kg^−1^, see Figure 5. The highest concentration of thorium was achieved for G10 granite: 189 Bq·kg^−1^ originating from Sweden, and the lowest for Indian granite G9: 5.5 Bq·kg^−1^. According to the data presented in the European Commission report [25], the average concentration of thorium isotope in the Earth’s crust is at a level of 40 Bq·kg^−1^. This means that all analyzed granites, except for G9 granite, contain higher activity concentrations of thorium isotope in relation to the mean content of this isotope in the earth’s crust, compare the concentration values on Figure 5 to the straight line representing the recommended value. In Poland, radioactivity analyses for Izerski granites were performed by Malczewski and Sitarek, see Table 2, [11].

In addition, Table 2 presents the average results of the specific activity concentration of natural radioisotopes in granites studied by other scientists, both in Poland and worldwide. 

Analyzing average specific activity concentrations of ^40^K obtained in this work with literature data, it can be seen that the results obtained by other scientists for granites from China, Finland, Spain and Sweden were similar to the granites analyzed in the scope of this research. The greatest difference in the specific activity concentrations of the potassium isotope was found for granite from India. The results obtained in this work differed by almost an order of magnitude in relation to the results obtained by other laboratories in the world. The analyzed Indian granite turned out to be the least radioactive, but as seen, this does not mean that all granites from this region contain low concentrations of this radionuclide. In case of radium ^226^Ra and thorium ^232^Th isotopes (see Table 1 and Table 2.), they are in most cases similar to the results obtained by other laboratories. Nevertheless, there are relatively large differences in the specific activity concentration of these isotopes, although the granites come from the same country, but they are different types of granites. For granites of Polish origin only the results obtained for Izerski granite could be compared with the results obtained by Malczewski and Sitarek [11]. In this case the specific activity of ^40^K obtained in this work was 30% higher than the value presented by Malczewski and Sitarek. While the specific activity of radium ^226^Ra was almost 10 times higher than specific activity concentration presented by Malczewski and Sitarek [11]. It can be assumed that the samples analyzed in this work may come from other, e.g., deeper layers, hence the difference.

Based upon the obtained specific activity concentration of the natural radioactive isotopes, the following parameters were calculated:

(1) Absorbed gamma dose rate D,
(2) Annual effective dose rate AED,
(3) Radium equivalent activity Ra_eq_,
(4) Internal hazard index H_in_,
(5) External hazard index H_ex_,
(6) Gamma index I,
(7) Alpha index I_α_.

The determined parameters are used in radiological protection to assess human exposure to ionizing radiation from natural radioactive isotopes i.e., thorium, radium and potassium. Table 3 contains the values of D, AED, Re_eq_, H_ex_, H_in_ I and I_α_, respectively.

In addition, statistical methods were applied, among which were: box-whisker charts, histograms, normality tests at 95% confidence level, outlier discard, and tests for correlations. The dependency of specific activity concentrations was searched between:

(1) ^40^K and ^226^Ra,
(2) ^40^K and ^232^Th,
(3) ^232^Th and ^226^Ra,
  and some conclusions, based on the statistical approach, were drawn.

**Figure 6:**
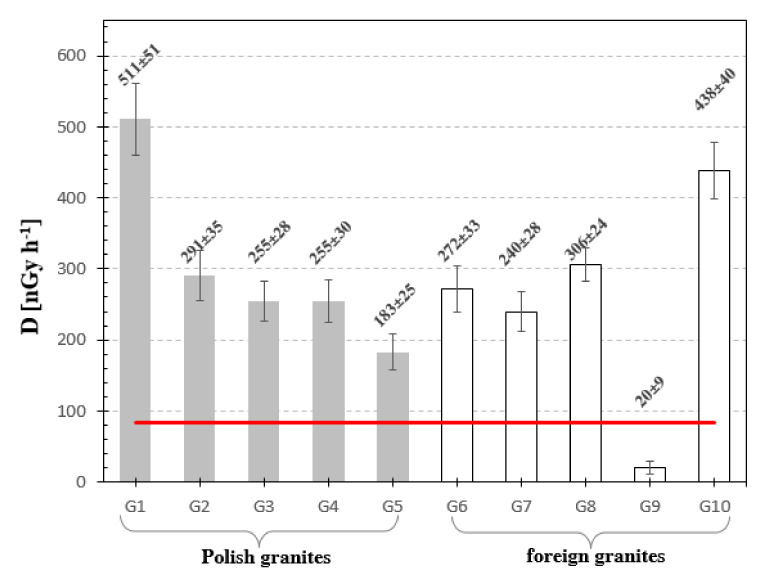
Absorbed gamma dose rate for analyzed samples. The horizontal line indicates the recommended value 84 nGy/h [22] for natural stones.

**Figure 7:**
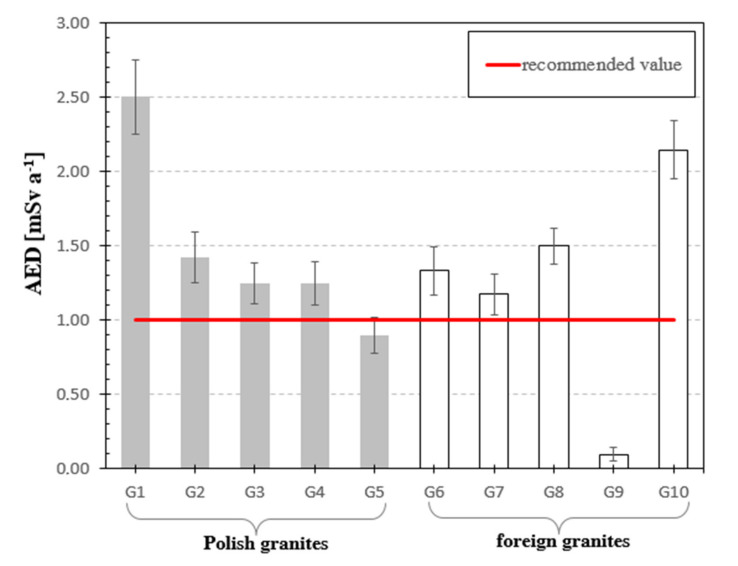
Annual effective dose for analyzed granite samples. The horizonal line indicates the recommended value of AED equal to 1 mSv per annum [22] for natural stones.

**Figure 8:**
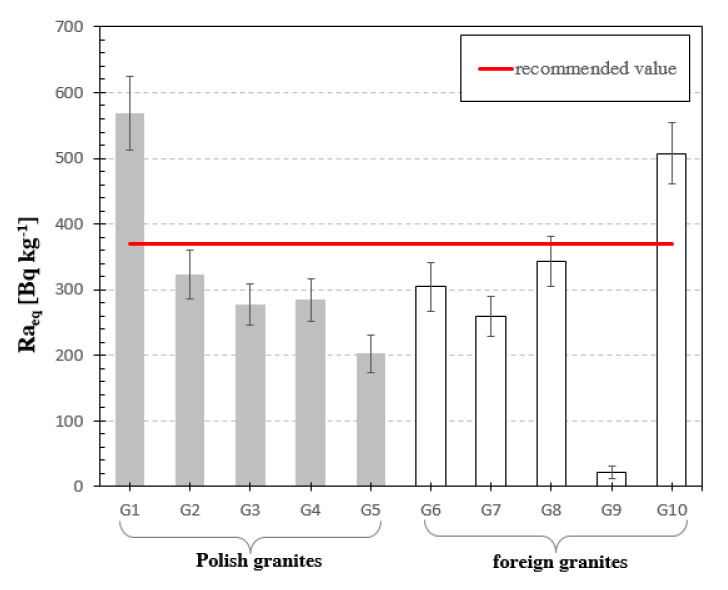
Radium Equivalent Activity in analyzed granite samples. The horizonal line indicates the recommended value of Ra_eq_ equal to 370 Bq/kg [23] for natural stones.

**Figure 9:**
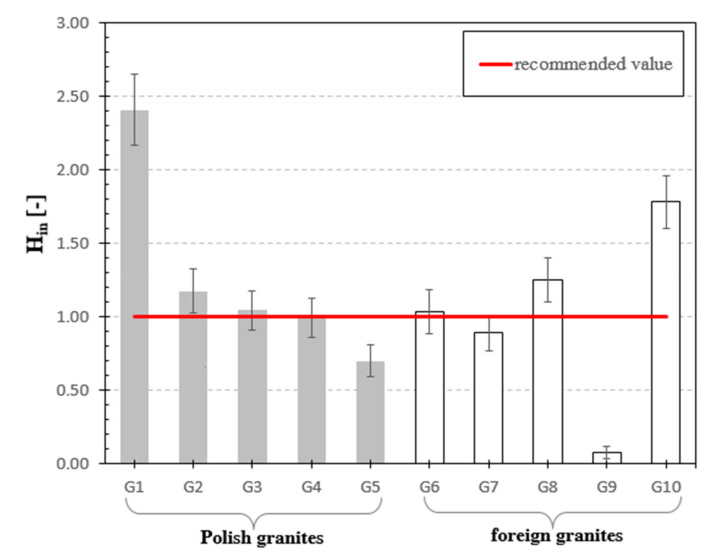
Internal Hazard Index for analyzed granite samples. The horizonal line indicates the recommended value of H_in_ equal to 1 [24] for natural stones.

**Figure 10:**
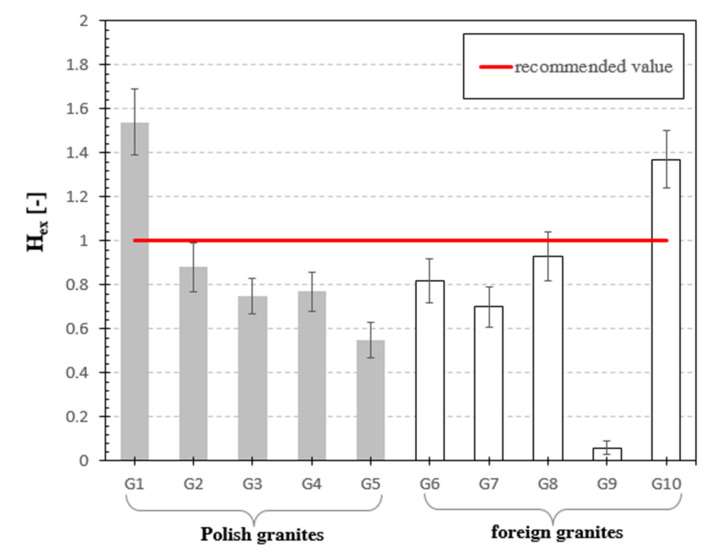
External Hazard Index for analyzed granite samples. The horizonal line indicates the recommended value of H_ex_ equal to 1 [24] for natural stones.

**Figure 11:**
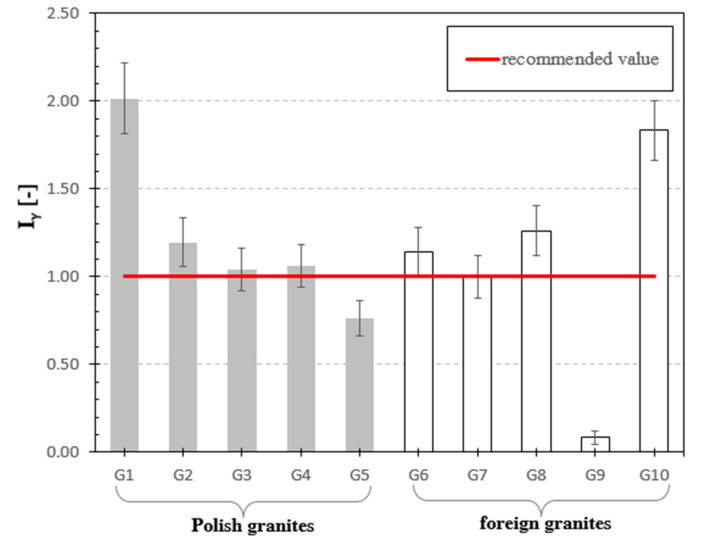
Gamma index for analyzed granite samples. The horizonal line indicates the recommended value of I_γ_ equal to 1 [24] for natural stones.

**Figure 12:**
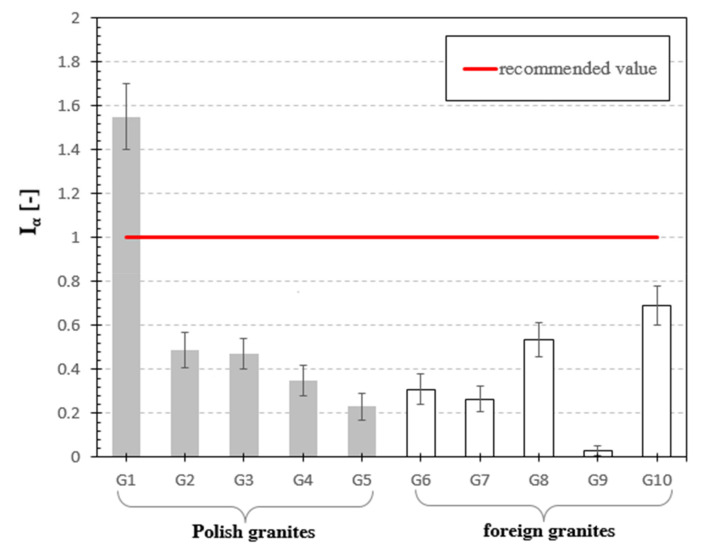
Alpha index for analyzed granite samples. The horizonal line indicates the recommended value of I_α_ equal to 1 [24] for natural stones.

Figure 6, Figure 7, Figure 8, Figure 9, Figure 10, Figure 11 and Figure 12 are the graphical representation of Table 3 radiological parameters. They clearly show that in many cases the parameters exceed recommended values [16,17,18,19] (red line on each chart). Figure 6 clearly shows that for almost every granite sample the recommended value of the absorbed gamma dose rate is exceeded.

Based upon the calculations made, it can be seen that the absorbed dose rate is in the range of 20 nGy h^−1^ to 511 nGy·h^−1^, Figure 6 and Table 3. The highest absorbed gamma dose rate was achieved for Izerski granite sample G1 and Swedish Vanga granite G10. For G9 granite the absorbed gamma dose rate was three times lower than the UNSCEAR 2000 recommended value [22]. In case of G1 and G10 granites the absorbed gamma dose rate recommended value was exceeded 6 times for G1 and over 5 times for G10. Only the G9 sample is considered safe, according to [22], for people and the environment.

The next analyzed parameter was annual effective dose rate (AED). The limit value of this parameter for all analyzed granite samples except G9 exceeded the value equal to one considered safe for people and the environment as referred to in Table 3, see Figure 7.

The annual effective dose rate was in the range of 0.12–2.50 mSv a^−1^. According to the UNSCEAR 2000 Report data [22] the results of the calculated doses shall not exceed 1 mSv·a^−1^. Considering the calculated values of radiological parameters, it can be stated that all granites except for Indian G9 granite exceeded the recommended value, and one granite of Polish origin G5 within the measurement error limit has a maximum value 1 mSv·a^−1^, see Figure 7. Most granite samples exceeded the limit value of 1 for AED: G2, G3, G4, G6, G7 and G8, and for two granite samples G1 and G10 the excess of AED was twofold. 

The next chart shows the values of radium equivalent activity Ra_eq_ [Bq·kg^−1^] for analyzed samples of granite, Figure 8. 

Radium equivalent activity of tested granite samples is within the range of 22 Bq·kg^−1^ to 570 Bq·kg^−1^. The highest Ra_eq_ was achieved for Izerski granite sample G1, and slightly lower for granite from Sweden G10. Only in these two samples the value of the radium equivalent activity exceeds the limit given in UNSCEAR 1982 Report [23], which is 370 Bq kg^−1^ and corresponds to annual effective dose of 1.0 mSv from natural radioactive isotopes.

Internal hazard index was analyzed, see Figure 9. According to the data presented in UNSCEAR 2008 Report [24] its value shall not exceed 1. For the analyzed materials this indicator was in the range from 0.08 to 2.42. Most of the measured granite samples exceeded this value, however, significant exceedances were recorded for granite samples G1, G8 and G10. In samples G5 and G9, the value of H_in_ was lower (in the case of G9 significantly lower), and in other samples this value oscillated within the boundaries of specified measurement uncertainty. 

The next analyzed parameter was the external hazard index. According to the data presented by UNSCEAR 2008 [24], its value should not exceed 1. For the analyzed materials, H_ex_ ranged from 0.06 to 1.54. The highest value of this indicator was reached for the sample of Izerski granite G1 and it was 54% higher than the reference value provided by UNSCEAR 2008. In two of the ten tested samples, the limit value of the external hazard index was exceeded, and for seven samples the external exposure index fluctuated within 55–93% of the recommended value.

The penultimate analyzed parameter from the point of view of radiological protection is the gamma index. The value of this index in the seven analyzed granites exceeded the safe reference value determined by European Commission [25], which is 1. The highest threat resulting from gamma radiation was recorded for samples of Izerski granite G1, which was 2.0, and for Swedish granite G10 at 1.84, respectively. The remaining part of the samples, i.e., G2, G3, G4, G6 and G8, exceeded the values up to 26% of the recommended value. Only the granite samples G5, G7 and G9 did not exceed 1, and G9 reached the lowest value (0.08), see Table 2 and Figure 11. 

The last parameter analyzed was the alpha index. The value of this parameter for only one sample of Izerski granite G1 was higher than 1 and exceeded the value given by Xinwei L. [26] and Tufail M. [27]. The values of the alpha indices ranged from 0.03 to 1.56, and seven of the remaining samples did not exceed the threshold of 50% of the recommended value, see Table 3 and Figure 12. 

## 6. Statistical Analysis

**Figure 13:**
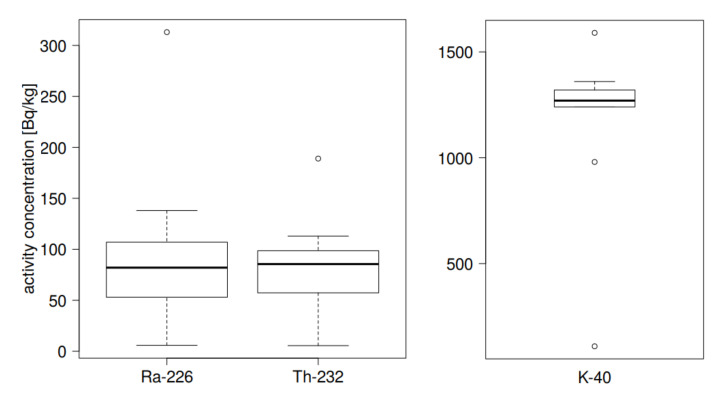
Box-whiskers charts for specific activity concentration of radium and thorium. Outliers that should be rejected in further analysis are clearly visible.

In order to estimate the relationship between the determined activity concentrations of radioactive isotopes—radium, thorium and potassium—a statistical analysis of the samples had to be carried out. It should be noted that a simple box-whiskers chart shows that for thorium and radium the largest values should be considered as the most outlying, while for potassium—the smallest and largest values, see Figure 13. Outliers may affect the proper interpretation of the statistical relationships, so they are usually discarded in further analysis, see e.g., [31,32,33]. However, first thing to do is to verify whether radium, thorium and potassium activities are normally distributed. This is a typical approach if one wish to perform further statistical analysis. In this study we take the Shapiro-Wilk test, which is one of the statistical tests for the verification of normality [31,32], and the adopted level of significance is (1 − α) × 100% = 95%. It turns out that for thorium the hypothesis about the normality of the distribution both in the full set of ten measurements and set without outliers (minimum value of 189 ± 14 [Bq kg^−1^]) cannot be rejected, whereas for radium—only when the largest value from the measurement set is rejected, see Table 3. In the case of potassium, only the rejection of the smallest and largest values ensured the normality of the distribution, however, due to the considerable reduction in the number of measurements, it was decided to leave the smallest value, especially since it neither affected Pearson correlation tests nor had significant influence on the determination factor which describes the model fitting quality. 

**Figure 14:**
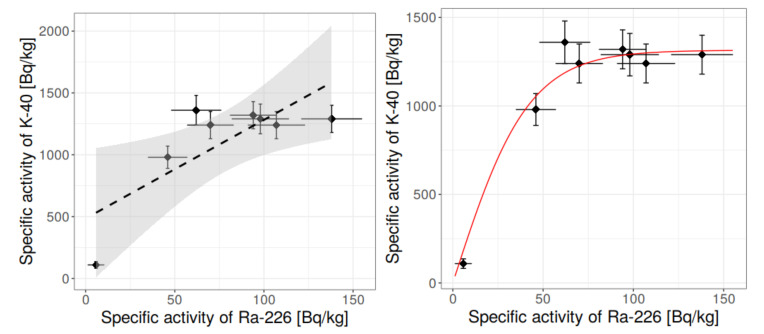
Relationship between specific activity concentration of ^40^K and ^226^Ra in measured granite samples, taking into account the rejection of outliers. Left: linear model with 95% confidence corridor, right: more realistic sigmoidal model (saturation for high concentration of radium). Determination factor: R^2^ = 0.92.

**Figure 15:**
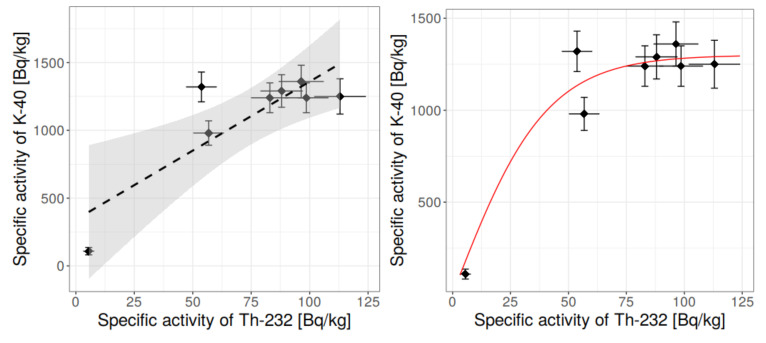
Relationship between specific activity concentration of ^40^K and ^232^Th in measured granite samples, taking into account the rejection of outliers. Left: linear model with 95% confidence corridor, right: more realistic sigmoidal model (saturation for high concentration of thorium). Determination factor: R^2^ = 0.90.

**Figure 16:**
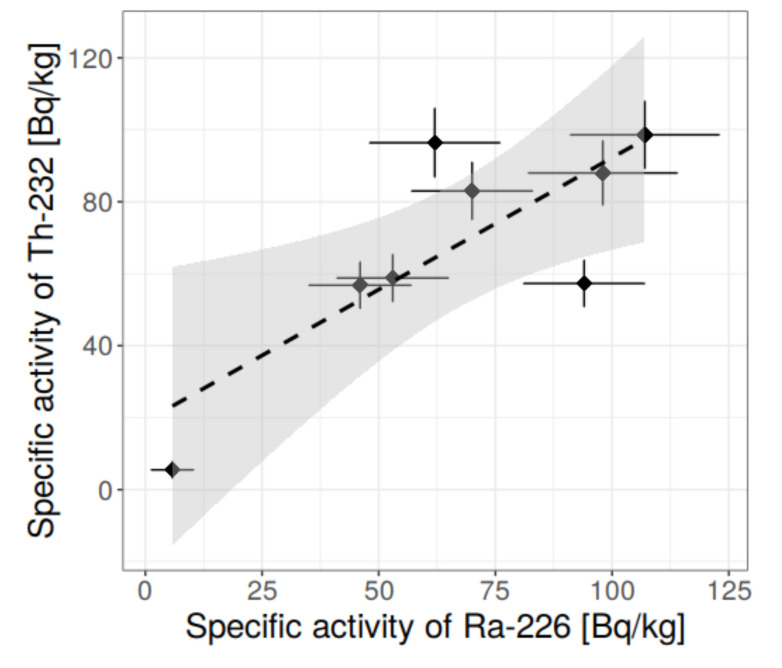
Relationship between specific activity concentration of ^232^Th and ^226^Ra in measured granite samples, taking into account the rejection of outliers. Clearly visible linear dependency. Fitted line is shown with 95% confidence corridor for a and b coefficients in the linear equation.

Therefore, having the results of the Shapiro-Wilk tests, Table 4, and knowing what values should be rejected in further analysis, it was possible to make the conclusion on the correlation for the binary systems radium-thorium, radium-potassium and thorium-potassium (for more on correlation and regression please refer to [34]). The relevant charts are presented in Figure 14, Figure 15 and Figure 16.

Figure 14 shows the relationship between potassium and radium. The chart does not include outliers that were rejected based on previous analysis. The simplest linear relationship gives a wide confidence corridor and a relatively small determination factor R^2^ ≈ 0.60. A much better model is the sigmoidal model, in which the initial linear growth is followed by saturation for higher values of radium specific activity concentration. Such approach seems to be justified at least for materials of natural origin where—in wide extent—less radioactive samples contain low concentrations of both potassium and radium, and more radioactive samples have higher specific activity concentrations. Thus, the initial linear growth is anticipated. However, potassium and radium are not connected neither by common series nor by their progenies, hence for certain specific activity concentration values distinct relationship between concentration of ^40^K and ^226^Ra cannot be expected. In other words, further increase in radium concentration should not affect potassium concentration, which is visible on the chart. The determination factor for the sigmoid model (hyperbolic tangent type) exceeded 0.9, thus the model is statistically valid. 

The relationship between the specific activity concentration of thorium and specific activity concentration of potassium is similar, Figure 15. The linear model is statistically better (determination factor R^2^ = 0.75), but the saturation for high concentration of thorium is clearly seen. The argumentation is similar to the radium–potassium relationship; therefore, the sigmoidal model was fitted, and the determination factor was 0.9. However, it is worth noting that essentially the sharp drop for low concentration is due only to one point; thus, to obtain higher credibility on the relationship for low specific activity concentration of thorium and potassium, the higher number of samples should be measured. In such case the statistical reasoning would be more significant. 

Thorium and radium show definitely stronger dependency, not only having higher determination factor for linear model, but also higher correlation coefficient (the correlation matrix is presented in Table 5). The correlation coefficient for radium-thorium is 0.84. The linear model can be adopted with good approximation, which is presented in Figure 16. Table 5 includes 90% confidence interval for correlation coefficient, and additional Pearson tests indicate that for 95% confidence level the hypothesis on ^226^Ra–^232^Th correlation higher than 0.8 cannot be rejected. 

One could wonder whether the above statistical analysis is sufficient since the total number of experiments is quite low. First of all, we have to be aware that the test on every sample was being performed for a long time (8 h). In some other articles the authors performed much shorter experiments on a few samples from one supplier, 1 h in [16] or even less in [35], then the resulting value was just a mean. Radioactivity is a physical quantity which is obtained from gamma ray spectrum. The longer experiment, the more accurate spectrum and thus better assessment of the radioactivity concentration. Typical time e.g., for soil radioactivity measurements varies from several up to 72 h, see [20,36,37,38]. According to the ergodic hypothesis, time integral for a function representing a physical quantity equals the integral over many configurations (here: samples) [39]. We believe the assumptions of the ergodic hypothesis are satisfied, providing good homogeneity for radionuclides distribution at a given place of extraction or production (with constant control of the ingredients content, as well).

## 7. Conclusions

The research of granites both Polish and foreign are important because they allow to determine the specific activity concentration of the radioactive isotopes of potassium, radium and thorium, which are used in housing, road and monumental (lapidary) construction. Granites have very good technical and chemical properties and are valued primarily for their aesthetic qualities. They are one of the raw materials whose intensive extraction does not limit the availability of its resources. Due to their properties, these rocks are widely used in construction industry and stonemasonry. Demand for aggregates crushing from rocks, such as granites, basalts and carbonate rocks, will certainly not decrease. Also, the increase in concrete production increases the consumption of cement and aggregate. Therefore, obtaining aggregates from existing mines as well as newly opened ones is becoming an urgent challenge for concrete producers. It should be taken into account that the natural radioactivity of rocks and minerals is a phenomenon occurring naturally and conventionally in the environment. Granite aggregates due to their properties are very widely used in bridge, road, water and mineral construction as structural elements. Therefore, there is a need for constant monitoring of their physical and chemical properties, one of which is radioactivity. 

The measurement results show that average specific activity concentration of ^226^Ra, ^232^Th and ^40^K for some granites are even several times higher than the average values recommended by the European Commission (40, 30 and 400 Bq·kg^−1^, respectively). Only granite G9, originating from India is characterized by low average concentration of these isotopes, and therefore is the safest for construction industry, taking into account potential radiological exposure.

The radiological parameters calculated during the research, such as absorbed gamma dose rate D, annual effective dose rate AED, radium equivalent activity Re_eq_, external hazard index H_ex_ and gamma index I_γ_, as well as alpha index I_α_ are—for the analyzed granites-higher than levels considered safe and given in UNSCEAR Reports [22,23,24] and others [27,28,29,30]. The absorber gamma dose rate and annual effective dose was exceeded for all granites except sample G9 (India). 

Admissible activity levels of radium equivalent activity and external hazard index were exceeded for two granites: G1 (Izerski) and G10 (Swedish). The internal hazard index was exceeded for most of the analyzed granite samples. Gamma index was exceeded for seven of ten samples: except G5 (Poland—Strzelin), G7 (Finland) and G9 (India) One sample of granite (G1—Izerski) exceeded alpha index. The use of granite (almost all samples) in housing is associated with an increased risk of exposure to ionizing radiation. 

The research shows that almost all granite samples can be associated with elevated radiological risk. The most significant is Polish Izerski granite, exceeding all radiological parameters, the following having the potential negative impact on human health is Swedish granite (sample G10). The remaining samples also exceed the levels of some radiological parameters according to their specific activity concentration of natural isotopes. According to UNSCEAR and European Commission recommendations, the safest granite came from India—it obtained the increased result only for internal hazard index. However, although radioactivity exceeds the standard levels in the above-mentioned cases, a suggestion that granites could be harmful or dangerous for users, goes far beyond the scope of this study. Some authors even point to the need of reconsider the limits for the radiological indicators recommended by UNSCEAR or European Comission [40]. Nonetheless, we live in natural radioactive background and we should be aware of it and monitor the quality and magnitude of this background.

The statistical analysis, in turn, suggests some kind of correlation between the specific activity concentration of radium and thorium in natural materials such as granites. The more thorough statistical analysis would be performed, if getting low activity samples. In this context, interesting is non-normality distribution of the radioactivity, as well. 

To conclude briefly:

(1) Granites are natural rocks used in stonemasonry, road construction, equipment in dwellings, cemetery monuments etc.
(2) Granites extracted from various places worldwide may possess increased radioactivity and thus higher radiation protection indicators.

## Figures and Tables

**Table 1:** The activity concentration of the natural isotopes of potassium ^40^K, radium ^226^Ra and thorium ^232^Th taking into account the uncertainty estimated by dedicated software.

Granites					
Sample	Poland		^40^K [Bq kg^−1^]	^226^Ra [Bq kg^−1^]	^232^Th [Bq kg^−1^]
G1	Izerski		1250 ± 130	313 ± 30	113 ± 11
G2	Karkonoski		1290 ± 120	98 ± 16	88 ± 9
G3	Siedlimowice		1320 ± 110	94 ± 13	57.3 ± 6.5
G4	Strzegom		1240 ±110	70 ± 13	83 ± 8
G5	Strzelin		980 ± 90	45.75	56.8 ± 6.5
**Sample**	**World**		**^40^K [Bq kg^−1^]**	**^226^Ra [Bq kg^−1^]**	**^232^Th [Bq kg^−1^]**
	**Country**	**Name/Color**			
G6	China	Royal Brown (beige)	1360 ± 120	62 ± 13	96.4 ± 9.6
G7	Finland	Baltic Green (green)	1590 ± 120	53 ± 12	58.7 ± 6.6
G8	Spain	Crema Julia (beige)	1240 ± 110	107 ± 16	98.6 ± 9.4
G9	India	Star Galaxy (black)	109 ± 27	5.8 ± 4.6	5.5 ± 2.3
G10	Sweden	Vanga (red)	1290 ± 110	138 ± 17	189 ± 14
**Recommended Values**			-	-	-
European Commission [25]			<400	<40	<40

**Table 2:** Comparison of obtained research results with literature data.

Granites					
Poland		^40^K [Bq kg^−1^]	^226^Ra [Bq kg^−1^]	^232^Th [Bq kg^−1^]	Source
Izerski		1246	312	113	Own material
		877	32	-	Malczewski, Sitarek (2005) [11]
Karkonoski		1293	98	88	Own material
Siedlimowice		1321	94	57	Own material
Strzegom		121	70	83	Own material
Strzelin		976	46	57	Own material
**World**		**^40^K [Bq kg^−1^]**	**^226^Ra [Bq kg^−1^]**	**^232^Th [Bq kg^−1^]**	**Source**
**Country**	**Name/Color**				
China	Royal Brown (beige)	1356	62	96	Own material
	Brown/beige	1152	77	78	J.H.Al-Zahrani (2017) [28]
	Grey	1122	102	123	Chen & Lin (1996) [29]
Finland	Baltic Green (green)	1590	53	59	Own material
	Brown	1343	131	217	Chen & Lin (1996) [29]
	Balmoral	1592	170	354	S. Pavlidou(2006) [30]
	Baltic Brown	1350	50	57	S. Pavlidou (2006) [30]
Spain	Crema Julia (beige)	1241	107	99	Own material
	Light red	1289	80	123	Chen & Lin (1996) [29]
	Extremadura	1293	101	48	Guillén (2014) [8]
	Blanco crystal	1190	163	91	S. Pavlidou (2006) [30]
	Gris Perla	1340	70	43	S. Pavlidou (2006) [30]
India	Star Galaxy (black)	109	5.8	5.5	Own material
	Green	LLD	7.7	1.2	J.H.Al-Zahrani (2017) [28]
	Dark red	1154	160	196	Chen&Lin (1996) [30]
	Grey	916	68	150	Chen & Lin (1996) [29]
	Multicolour	926	11	84	S. Pavlidou (2006) [30]
Sweden	Vanga (red)	1294	138	189	Own material
	Brown	1227	111	101	Chen & Lin (1996) [29]

**Table 3:** Absorbed gamma dose rate, annual effective dose rate, radium equivalent activity, external and internal hazard indices, gamma index and alpha index. Values and calculated uncertainties are presented.

No.	D [nGy h^−1^]	AED [mSv a^−1^]	Ra_eq_ [Bq kg^−1^]	H_ex_ [-]	H_in_ [-]	I [-]	I_α_ [-]
G1	511 ± 51	2.50 ± 0.25	570 ± 56	1.53 ± 0.15	2.41 ± 0.24	2.0 ± 0.2	1.56 ± 0.15
G2	291 ± 35	1.43 ± 0.17	324 ± 38	0.87 ± 0.11	1.18 ± 0.15	1.20 ± 0.14	0.49 ± 0.08
G3	255 ± 28	1.25 ± 0.14	277 ± 31	0.75 ± 0.08	1.05 ± 0.13	1.04 ± 0.12	0.47 ± 0.07
G4	255 ± 30	1.25 ± 0.15	284 ± 33	0.77 ± 0.09	1.00 ± 0.13	1.06 ± 0.12	0.35 ± 0.07
G5	183 ± 25	0.90 ± 0.12	202 ± 28	0.55 ± 0.08	0.70 ± 0.11	0.76 ± 0.10	0.23 ± 0.06
G6	272 ± 33	1.33 ± 0.16	304 ± 37	0.82 ± 0.10	1.03 ± 0.15	1.14 ± 0.14	0.31 ± 0.07
G7	240 ± 28	1.18 ± 0.14	259 ± 31	0.70 ± 0.09	0.89 ± 0.12	1.00 ± 0.12	0.26 ± 0.06
G8	306 ± 24	1.50 ± 0.12	343 ± 38	0.93 ± 0.11	1.26 ± 0.15	1.26 ± 0.14	0.53 ± 0.08
G9	20 ± 9	0.10 ± 0.04	22 ± 10	0.06 ± 0.03	0.08 ± 0.04	0.08 ± 0.04	0.029 ± 0.023
G10	438 ± 40	2.15 ± 0.20	508 ± 46	1.37 ± 0.13	1.78 ± 0.18	1.84 ± 0.17	0.69 ± 0.09
UNSCEAR 2000 UNSCEAR 1982 European Commission	≤84	≤1	≤370	≤1	≤1	≤1	≤1

**Table 4:** Shapiro-Wilk test results for potassium, radium and thorium concentration activity measurements.

^40^K Sample	Shapiro-Wilk Test Results	Conclusion
G1.G2.G3.G4.G5.G6.G7.G8.G9.G10 (Full set of measurements)	W= 0.693 p = 0.00073 < 0.05	Hypothesis of normal distribution shall be rejected
Without G7	W = 0.597 p = 0.000088 < 0.05	Hypothesis of normal distribution shall be rejected
Without G7 and G9	W = 0.88 p = 0.157 > 0.05	Hypothesis of normal distribution shall not be rejected
**^226^** **Ra Sample**	**Shapiro** **-** **Wilk Test Results**	**Conclusion**
G1.G2.G3.G4.G5.G6.G7.G8.G9.G10 (full set of measurements)	W = 0.794 p = 0.012 < 0.05	Hypothesis of normal distribution shall be rejected
Without G1	W = 0.985 p = 0.984 > 0.05	Hypothesis of normal distribution shall not be rejected
**^232^** **Th Sample**	**Shapiro** **-** **Wilk Test Results**	**Conclusion**
G1.G2.G3.G4.G5.G6.G7.G8.G9.G10 (Full set of measurements)	W = 0.922 p = 0.37 > 0.05	Hypothesis of normal distribution shall not be rejected
Without G10	W = 0.909 p = 0.135 > 0.05	Hypothesis of normal distribution shall not be rejected
Without G1 and G10	W = 0.866 p = 0.14 > 0.05	Hypothesis of normal distribution shall not be rejected

**Table 5:** Correlation matrix for specific activity concentration of ^40^K, ^226^Ra and ^232^Th with 90% confidence interval for ρ.

	^40^K		^226^Ra		^232^Th	
**^40^** **K**	X		0.78	0.30 < ρ < 0.94	0.84	0.46 < ρ < 0.94
**^226^** **Ra**	0.78	0.30 < ρ < 0.94	X		0.79	0.33 < ρ < 0.94
**^232^** **Th**	0.84	0.46 < ρ < 0.94	0.79	0.33 < ρ < 0.95	X	

## References

[1] Kryza R., Walendowski H. (2007). Very Good Polish Granites.

[2] Majerowicz A., Wierzchołowski B. (1990). Petrology of Igneous Rocks.

[3] Ryka W., Kozłowski K. (1981). Petrography of Igneous Rocks.

[4] Maślankiewicz K. (1957). Introduction to Rock Science.

[5] Maślankiewicz K. (1973). Among Minerals and Rocks.

[6] Wichrowska M., Wichrowski Z., Żejmo I. (1972). Natural radioactivity of biotytes from Strzegom and Strzelin granitoides. Kwartalnik Geologiczny.

[7] Mason B., Moore C.B. (1982). Principles of Geochemistry.

[8] Uosif M., Issa S.A., El-Salam L.A. (2015). Measurement of natural radioactivity in granites and its quartz-bearing gold at El-Fawakhir area (Central Eastern Desert), Egypt. J. Radiat. Res. Appl. Sci..

[9] Pieńkowski S., Rygierowa D., Szwacka C.J., Twardowska B., Zmysłowska S. (1956). Types of distribution of radioactive substances in Polish rocks. Arch. Miner..

[10] Żejmo I., Wichrowski Z. (1969). Radioactivity of Pliocene clays from Konin. Acta Geophys. Pol..

[11] Malczewski D., Sitarek A., Żaba J., Dorda J. (2005). Natural radioactivity of selected cristalline rocks of Izerski block. Przegląd Geologiczny.

[12] Eisenbud M., Gesell T.P. (1997). Environmental Radioactivity from Natural, Industrial, and Military Sources.

[13] Plewa M., Plewa S. (1992). Petrophysics.

[14] Council Directive 2013/59/Euratom of 5 December 2013 Laying Down Basic Safety Standards for Protection against the Dangers Arising from Exposure to Ionising Radiation, and Repealing Directives 89/618/Euratom, 90/641/Euratom, 96/29/Euratom, 97/43/Euratom and 2003/122/Euratom. https://eur-lex.europa.eu/eli/dir/2013/59/oj.

[15] Papastefanou C., Stoulos S., Manolopoulou M. (2005). The radioactivity of building materials. J. Radioanal. Nucl. Chem..

[16] Xhixha G., Ahmeti A., Bezzon G.P., Bitri M., Buso G.P., Callegari I., Cfarku F., Colonna T., Massa G., Mou L. (2013). First characterisation of natural radioactivity in building materials manufactured in Albania. Radiat. Prot. Dosim..

[17] Ademola A.K., Abodunrin O.P., Fatai M.A., Omoboyede J.O. (2017). Assessment of natural radioactivity levels in cement samples commonly used for construction in Lagos and Ogun states, Nigeria. Elixir Nucl. Radiat. Phys..

[18] Róg L., Wawrzynkiewicz W., Hamala K., Rompalski P., Solik M. (2008). Determining of accuracy of drawing, preparation and analysis of biofuels samples and fuels of waste origin. Res. Rep. Min. Environ..

[19] Barnekow U., Fesenko S., Kashparov V., KisBenedek G., Matisoff G., Onda Y., Sanzharova N., Tarjan S., Tyler A., Varga B. (2019). Guidelines of Soil and Vegetation Sampling for Radiological Monitoring.

[20] Nkoulou J.E.N., Talla S.F., Bineng G.S., Manga A., Siaka Y.F.T., Saïdou (2018). Natural Radioactivity Measurements in Soil, External Dose and Radiological Hazard Assessment in the Uranium and Thorium Bearing Region of Lolodorf, Cameroon. Radioisotopes.

[21] Analysis of Natural Radionuclide Concentrations in Selected Building Materials Available on the Domestic Market. https://www.shs-conferences.org/articles/shsconf/abs/2018/18/shsconf_infoglob2018_02003/shsconf_infoglob2018_02003.html.

[22] United Nations Scientific Committee on the Effects of Atomic Radiation (2000). Sources and Effects of Ionizing Radiation: Sources.

[23] United Nations Scientific Committee on the Effects of Atomic Radiation (1982). Ionizing Radiation, Sources and Biological Effects, United Nations Scientific Committee on the Effects of Atomic Radiation (UNSCEAR) 1982 Report.

[24] United Nations Scientific Committee on the Effects of Atomic Radiation (2008). Effects of Ionizing Radiation, United Nations Scientific Committee on the Effects of Atomic Radiation (UNSCEAR) 2006 Report.

[25] European Commission (1999). Radiation Protection Unit, Radiological protection principles concerning the natural radioactivity of building materials. Radiat. Prot..

[26] Xinwei L. (2004). Radioactivity level in Chinese building ceramic tile. Radiat. Prot. Dosim..

[27] Tufail M., Akhtar N., Javied S., Hamid T. (2007). Natural Radioactivity hazards of building bricks fabricated from soil of two districts of Pakistan. J. Radiol. Prot..

[28] Al-Zahrani J.H. (2017). Estimation of natural radioactivity in local and imported polished granite used as building materials in Saudi Arabia. J. Radiat. Res. Appl. Sci..

[29] Chen C.-J., Lin Y.-M. (1996). Assessment of building materials for compliance with regulations of ROC. Environ. Int..

[30] Pavlidou S., Koroneos A., Papastefanou C., Christofides G., Stoulos S., Vavelides M. (2006). Natural radioactivity of granites used as building materials. J. Environ. Radioact..

[31] Gupta A., Mishra P., Pandey C.M., Singh U., Sahu C., Keshri A. (2019). Descriptive statistics and normality tests for statistical data. Ann. Card. Anaesth..

[32] Das K.R., Imon A.H.M.R. (2016). A Brief Review of Tests for Normality. Am. J. Theor. Appl. Stat..

[33] Cousineau D., Chartier S. (2010). Outliers detection and treatment: A review. Int. J. Psychol. Res..

[34] Bewick V., Cheek L., Ball J. (2003). Statistics review 7: Correlation and regression. Crit. Care.

[35] Zalewski M., Tomczak M., Kapata J. (2001). Radioactivity of building materials available on northeastern Poland. Pol. J. Environ. Stud..

[36] Stobiński M., Szarlowicz K., Reczynski W., Kubica B. (2013). The evaluation of ^137^Cs radioactivities in soils taken from the Babia Góra National Park. J. Radioanal. Nucl. Chem..

[37] Kubica B., Stobiński M., Szaciłowski G., Szarlowicz K. (2017). The activity of selected gamma radionuclides in the Tatra National Park. E3S Web Conference.

[38] Vukašinović I., Todorović D., Životić L., Kaluđerović L., Đorđević A. (2017). An analysis of naturally occurring radionuclides and ^137^Cs in the soils of urban areas using gammaray spectrometry. Int. J. Environ. Sci. Technol..

[39] Moore C.C. (2015). Ergodic theorem, ergodic theory, and statistical mechanics. Proc. Natl. Acad. Sci. USA.

[40] Trevisi R., Risica S., D’Alessandro M., Paradiso D., Nuccetelli C. (2012). Natural radioactivity in building materials in the European Union: A database and an estimate of radiological significance. J. Environ. Radioact..

